# Oxygen regulation of microbial communities and chemical compounds in cigar tobacco curing

**DOI:** 10.3389/fmicb.2024.1425553

**Published:** 2024-07-23

**Authors:** Juan Yang, Fang Xue, Dongliang Li, Jiaowen Chen, Guiyang Shi, Guangfu Song, Youran Li

**Affiliations:** ^1^Key Laboratory of Industrial Biotechnology, Ministry of Education, School of Biotechnology, Jiangnan University, Wuxi, China; ^2^National Engineering Research Center for Cereal Fermentation and Food Biomanufacturing, Jiangnan University, Wuxi, Jiangsu, China; ^3^Jiangsu Provincial Engineering Research Center for Bioactive Product Processing, Jiangnan University, Wuxi, Jiangsu, China; ^4^Key Laboratory of Chinese Cigar Fermentation, Cigar Technology Innovation Center of China Tobacco, Tobacco Sichuan Industrial Co., Ltd, Chengdu, China

**Keywords:** cigar leaf, oxygen regulation, aging and fermentation, microbial diversity, chemical components

## Abstract

**Introduction:**

Curing is a critical process that determines the sensory quality of cigars. The impact of oxygen on cigar curing and the mechanisms by which it regulates microbial changes affecting cigar quality are not well understood.

**Methods:**

In this study, we selected handmade cigars from the same batch and conducted curing experiments in environments with varying oxygen concentrations (equivalent to 0.1%, 6–12, and 15% of atmospheric oxygen concentration). We collected samples over 60 days and analyzed the distribution of microbial communities using high-throughput sequencing. Combined with the analysis of total sugars, proteins, flavor substances, and other chemical compounds, we elucidated how different oxygen concentrations affect the cigar curing process, influence microbial community succession, and ultimately impact cigar quality.

**Results:**

Our results revealed significant differences in bacterial community composition under different oxygen conditions. Under aerobic conditions, *Cyanobacteria* were the dominant bacteria, while under oxygen-limited conditions, *Staphylococcus* and *Corynebacterium* predominated. As oxygen concentration decreased, so did the richness and diversity of the bacterial community. Conversely, oxygen concentration had a lesser impact on fungi; *Aspergillus* was the dominant genus in all samples. We also found that Enterococcus showed a positive correlation with aspartic acid, alanine, and 4-aminobutyric acid and a negative correlation with cysteine. Cigars cured at 15% oxygen concentration for 60 days exhibited optimal quality, particularly in terms of flavor richness and sweetness.

**Discussion:**

These findings suggest that oxygen concentration can alter cigar quality by regulating aerobic and anaerobic microbial community succession. The relationship between specific microbial communities and flavor compounds also provides a theoretical reference for developing artificial control technologies in the cigar curing process.

## 1 Introduction

Cigars are pure natural tobacco products hand-rolled from whole tobacco leaves and primarily consist of three parts: the wrapper, binder, and filler leaves (Sun et al., 2020). Cigars are noted for their rich aroma, full-bodied flavor, strong kick, low tar content, among other characteristics (Zhang et al., 2020). Unlike cigarettes, cigar leaves undergo a special aging and fermentation process after rolling; this involves storing the cigars in a controlled temperature and humidity environment to mature. Aging and fermentation aim to balance the moisture content of the cigar and promote the breakdown and transformation of internal substances to enhance sensory quality. This technique is a key factor in determining the sensory quality of cigars. Appropriate technical requirements for aging and fermentation are set based on cigar formula characteristics. Cigars that have undergone this process are characterized by harmonious aroma, prominent flavor, reduced harshness and irritants, and a comfortable aftertaste. Key factors affecting aging quality include environmental temperature, relative humidity, time, aging media, and oxygen concentration.

Typical conditions for cigar aging and fermentation include temperatures of 20–25°C, relative humidity of 60–75%, and varying aging durations depending on when cigars reach an optimal smoking state. Aging media, such as humidors, moisture boxes, and natural fragrant substances, can influence cigar flavor (Hu et al., 2022; Zhao et al., 2022; Ren et al., 2023). Song et al. (2018) found that after 21–27 months of aging at 70% ± 5% humidity and 18–27°C, the chemical composition, physical appearance, and sensory quality of cigar filler leaves improved. Huang et al. (2010) observed that the content of aroma-causing components (alcohols, aldehydes, ketones, acids, and esters) in tobacco leaves gradually increased with extended alcoholization time. While considerable research has focused on the effects of environmental temperature, humidity, aging time, and media on cigar quality during aging, the impact of oxygen concentration remains unclear. Liu et al. (2016) showed that an oxygen volume fraction of 50–60% and aging of about 40 days resulted in better re-cured tobacco quality. Yang et al. (2017) indicated that low-oxygen treatment could inhibit color darkening during tobacco leaf aging and extend the suitable quality period. Oxygen is crucial for normal fermentation and quality improvement of tobacco leaves. During aging, oxygen participates in chemical reactions and can effectively regulate the aging rate by changing the environmental oxygen content. Choosing an appropriate oxygen content can improve the aging rate, speed up inventory turnover, reduce maintenance costs, and ensure cigar quality and production continuity while maintaining tobacco leaf quality. Although oxygen concentration regulation plays an important role in the quality of tobacco leaf aging, its impact on cigar leaf quality during aging is still unclear. Additionally, microorganisms play a crucial role in the fermentation and aging process of cigars, and changes in microbial community succession can accelerate fermentation and improve quality. Zhang et al. (2018) used high-throughput sequencing and traditional isolation to compare and analyze the diversity and succession of bacteria on the surface of cigar wrapper raw materials during different fermentation periods, confirming that high-throughput sequencing can more comprehensively reveal microbial diversity and succession. Chen et al. (2023) found that the material solution could inhibit oleic acid content in cigar tobacco leaves, helping to reduce astringency and irritation, and that changes in chemical components would affect the overall smoking quality. Changes in oxygen concentrations cause microorganisms to migrate and affect the chemical composition of cigars, but the mechanism of action of microorganisms in this process is also unclear.

This study designed three different oxygen concentration conditions to investigate changes in microbial communities and chemical components of cigar leaves during the aging process. Using high-throughput sequencing technology, we studied changes in microbial communities under aerobic and oxygen-limited conditions during leaf aging. We systematically measured changes in nicotine, total nitrogen, total soluble sugars, monosaccharides, and organic acids in cigar leaves throughout the aging process. We analyzed the correlation between changes in leaf components and microbial community dynamics during this process and attempted to establish a relationship between microbial community dynamics and changes in leaf components. This will lay a foundation for further improving controllability of cigar leaf aging quality and leaf quality.

## 2 Materials and methods

### 2.1 Experimental materials

The DX-4 tobacco leaf samples used in this study were planted in Deyang (Sichuan, China), harvested in 2022. The samples were obtained after air-curing in a barn until the humidity of tobacco leaves reached 17–20%.

### 2.2 Methods

#### 2.2.1 Tobacco leaf fermentation treatment and sampling

Cigar tobacco leaves were evenly packed into anaerobic fermentation vessels and placed in a constant temperature incubator for fermentation experiments. The fermentation conditions are shown in Table 1. The absolute concentration of oxygen in the air is 21%, and the oxygen-limited conditions were controlled at oxygen concentrations of 0.1%, 6–12, and 15%. The oxygen-limited conditions of 0.1% and 6–12% were carried out in 2.5 L anaerobic fermentation vessels by placing AnaeroPack·Anaerobic and AnaeroPack·Microaerophilic gas-producing bags to control the oxygen concentration; the oxygen concentration of 15% was carried out in a vacuum incubator, by controlling the vacuum pump to achieve a vacuum of −0.2 bar. On days 0, 5, 10, 30, and 60 of fermentation, cigar leaves were sampled using the five-point sampling method, with a sampling amount of 5 cigarettes per point. The collected samples were named as shown in Table 1 and stored at −40°C in sterile sampling bags for analysis.

**Table 1:** Curing conditions for cigar tobacco leaves.

Serial number	Curing time (d)	Oxygen concentration	Temperature (°C)
C0	0	\	\
A1	5	0.1%	20
A2	10	0.1%	20
A3	30	0.1%	20
A4	60	0.1%	20
A5	5	0.1%	16
A6	10	0.1%	16
A7	30	0.1%	16
A8	60	0.1%	16
M1	5	6% ~ 12%	20
M2	10	6% ~ 12%	20
M3	30	6% ~ 12%	20
M4	60	6% ~ 12%	20
M5	5	6% ~ 12%	16
M6	10	6% ~ 12%	16
M7	30	6% ~ 12%	16
M8	60	6% ~ 12%	16
B1	5	15%	20
B2	10	15%	20
B3	30	15%	20

#### 2.2.2 Determination of conventional components in tobacco leaves

In accordance with standards YC/T 160–2002, YC/T 161–2002, YC/T 159–2002, YC/T 217–2007, and YC/T 162–2011, a continuous flow analyzer was used to determine the chemical components (nicotine, total nitrogen, water-soluble total sugar, reducing sugar, potassium, and chlorine) in tobacco leaves before and after fermentation (Rong et al., 2021). Each sample was measured in triplicate, and the average value was reported.

#### 2.2.3 Determination of flavor components in tobacco leaves

The composition and content of non-volatile organic acids in cigar tobacco leaves were determined using gas chromatography (GC) (Du et al., 2017). The cigar tobacco leaf samples underwent methylation using the methanol-sulfuric acid method: 1 g of cigar tobacco leaf powder was weighed into a 100 mL round-bottom flask, combined with 300 μL of succinic acid internal standard solution and 50 mL of a 5% sulfuric acid-methanol solution, and refluxed at 80°C for 2 h to obtain the methylated solution. Dichloromethane was then added and the mixture was shaken to obtain the extract, which was filtered through a 0.22 μm organic phase filter membrane for analysis. GC testing conditions included a DB-5MS chromatographic column; an injection volume of 1 μL with a flow rate of 1.5 mL/min; helium as the carrier gas; an injection port temperature of 280°C; and a temperature program that started at 40°C for 3 min, then increased to 280°C at a rate of 10°C/min and held for 30 min. Gas chromatography–mass spectrometry (GC–MS) was used to determine the composition and content of aroma components in cigar tobacco leaves. Quantification was performed using an internal standard method: 2 g of cigar tobacco leaf powder was placed into a 50 mL centrifuge tube, combined with 10 mL of deionized water, 10 mL of acetonitrile, and 50 μL of ethyl benzoate internal standard solution, shaken for 120 min, then treated with a QuEChERS extraction kit and shaken for an additional 2 min. After centrifugation, 1 mL of supernatant was transferred to a new centrifuge tube, mixed with 150 mg of anhydrous MgSO4, shaken for another 2 min, centrifuged again, and filtered; the filtrate was collected for GC–MS analysis. GC–MS conditions included a DB-5 MS chromatographic column; helium as the carrier gas with a flow rate of 1.5 mL/min; and a temperature program that started at 60°C and increased at a rate of 2°C/min to 250°C, then at a rate of 5°C/min to 290°C and held for 20 min.

#### 2.2.4 Sensory quality evaluation

The sensory quality—including mellowness, richness, miscellaneous gases, fullness, fluency, finesse, sweetness, irritation, sensation of recirculation, combustibility, grey tuff and balance—was evaluated by experts from the cigar industry according to the “YC/T 415–2011 Method for Tobacco Processing-Sensory Evaluation Methods” on a nine-point scale.

#### 2.2.5 High-throughput sequencing and data analysis of microorganisms

##### 2.2.5.1 Microbial genome DNA extraction, PCR amplification, and Illumina sequencing

After shredding the cigar tobacco leaves under aseptic conditions, they were placed in an adequate amount of sterile saline and shaken at 37°C at 180 rpm for 60 min. The mixed microbial community on the surface of the leaves was then obtained by centrifugation. The total DNA of the microbial community was extracted following the instructions of the E.Z.N.A.® soil DNA kit (Omega Bio-tek, U.S.), and the DNA quality, concentration, and purity were measured using a NanoDrop2000; the DNA was diluted with sterile water to an appropriate concentration (1 ng/μL). Using the diluted genomic DNA as a template, primers 338F (5’-ACTCCTACGGGAGGCAGCAG-3′) and 806R (5′-GGACTACHVGGGTWTCTAAT-3′) for bacterial genome DNA, and primers ITS5-1737F (5′-GGAAGTAAAAGTCGTAACAAGG-3′) and ITS2-2043R (5′-GCTGCGTTCTTCATCGATGC-3′) for fungal genome DNA, were selected according to the amplification system and method described by Apprill et al. to PCR amplify the V3-V4 region of the 16S rRNA gene and the ITS1 region of the fungal genome DNA, respectively (Langille et al., 2013). After purification, detection, and quantification of the PCR products, libraries were constructed using the NEXTFLEX Rapid DNA-Seq Kit, and sequencing was completed on the Illumina MiSeq PE300 platform.

##### 2.2.5.2 Data analysis

Bioinformatics analysis was mainly conducted on the BGI Genomics Cloud Platform,^1^ starting with merging PE reads obtained from sequencing using Flash software, followed by quality control and filtering of sequences with Fastp software to obtain valid sequences. Sequences were clustered into OTUs at a similarity level of 97% using Uparse software. RDP Classifier software was used to annotate species classification of OTUs representative sequences at a 97% similarity level, while Mothur software was employed to calculate biodiversity indices such as Chao1 and Shannon indices. QIIME software was used to generate species abundance tables at various taxonomic levels. Based on bioinformatics analysis, further analyses such as Venn diagrams of species, community composition analysis, Alpha diversity analysis, Beta diversity analysis, and species difference analysis were conducted using Microsoft Excel 2019 and Origin Pro 2021 software. PICRUSt functional prediction was performed using bacterial 16S rRNA sequencing data to determine the abundance of microbial functional genes in KEGG metabolic pathways; identified fungal OTUs were classified using the FUNGuild database.

## 3 Results and analysis

### 3.1 Microbial community composition

#### 3.1.1 Microbial community composition under oxygen-limited fermentation conditions

**Figure 1:**
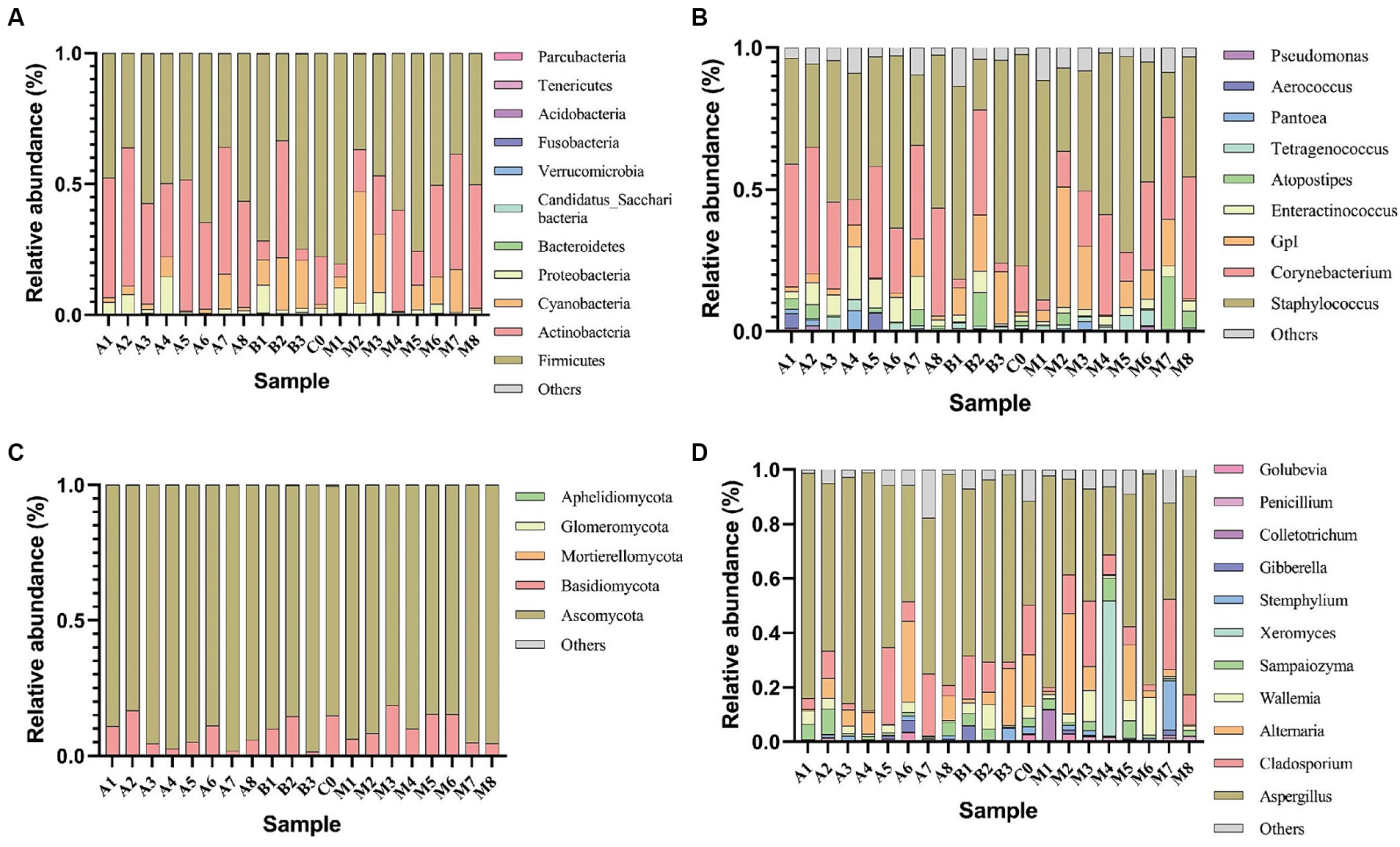
Relative abundance of major bacterial phyla **(A)** and genera **(B)**, as well as major fungal phyla **(C)** and genera **(D)** in cigar tobacco leaf samples under oxygen-limited conditions.

Illumina HiSeq high-throughput sequencing was performed on the microbial communities in 20 cigar tobacco samples, yielding 1,556,097 high-quality 16S rRNA sequences and 1,829,319 high-quality ITS sequences. Each sample produced at least 76,912 16S rRNA sequences and 84,575 ITS sequences, with an average of 77,805 16S rRNA sequences and 91,466 ITS sequences per sample. These sequences were taxonomically identified and displayed in the form of a histogram of species distribution as shown in Figure 1. The dominant bacterial phyla were *Firmicutes* and *Actinobacteria* (Figure 1A), and the dominant genera were *Staphylococcus* and *Corynebacterium* (Figure 1B). According to the literature (Song et al., 2022), the genus *Staphylococcus* has a relatively high abundance in cigar tobacco leaves, which is consistent with the results of this study. The genus *Staphylococcus* is key in the formation of flavor compounds (Hu et al., 2022), capable of metabolizing reducing sugars, malic acid, citric acid, etc., and is associated with the production of proteases and lipases, contributing to the generation of flavor compounds (Zheng et al., 2022). In fungi, the phylum *Ascomycota* was absolutely dominant, followed by *Basidiomycota* (Figure 1C); the genus *Aspergillus* was the most dominant (Figure 1D). There were certain differences in the relative abundance of microbes under different fermentation conditions. For example, the relative abundance of the dominant phylum *Firmicutes* and genus *Staphylococcus* ranged from 80.37 to 33.25% and from 77.42 to 15.97%, respectively. In fungi, the relative abundance of the dominant phylum *Ascomycota* and genus *Aspergillus* ranged from 98.44 to 81.38% and from 87.54 to 25.07%, respectively. As fermentation progressed, the microbial abundance of both *Firmicutes* and *Staphylococcus* showed a trend of initially decreasing and then increasing, with a decrease at the end of fermentation; while the microbial abundance of both *Ascomycota* and *Aspergillus* showed a trend of initially increasing and then decreasing, with an increase at the end of fermentation. The normal reproduction and metabolism of most microorganisms in *Ascomycetes* and *Aspergillus* require a certain oxygen concentration. Reducing the concentration of oxygen retards the growth of *Ascomycota* and *Aspergillus* (Northolt and Bullerman, 1982). Although microbial communities in cigar tobacco leaf samples under oxygen-limited conditions varied, the dominant genera were consistent and universal, mainly including *Staphylococcus*, *Corynebacterium*, and *Aspergillus*. The variation in fungal communities in cigar tobacco leaf samples under oxygen-limited conditions was less than that of bacteria.

#### 3.1.2 Microbial community composition under aerobic fermentation conditions

**Figure 2:**
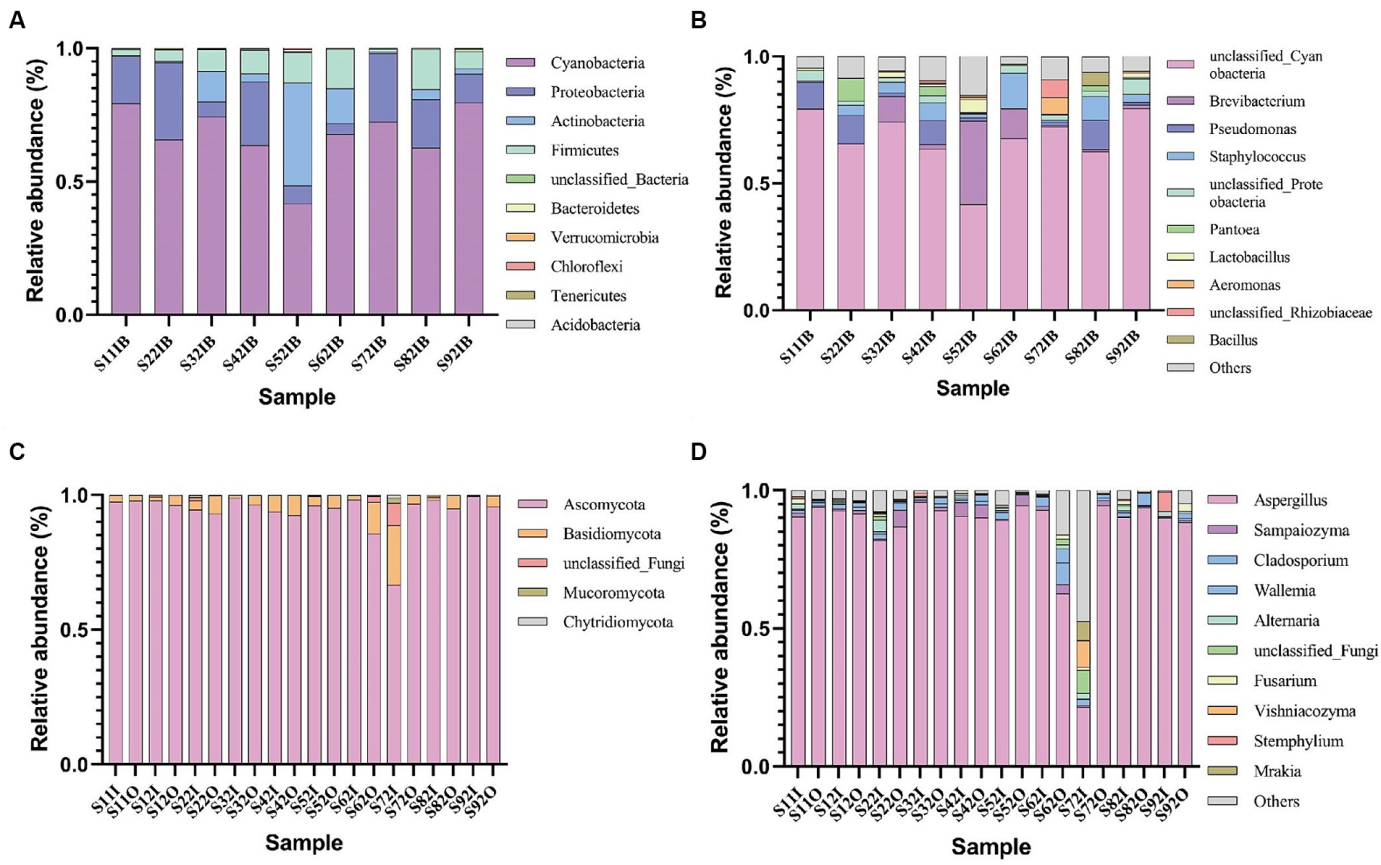
Relative abundance of major bacterial phyla **(A)** and genera **(B)**, as well as major fungal phyla **(C)** and genera **(D)** in cigar tobacco leaf samples under aerobic conditions.

As shown in Figure 2, under aerobic fermentation conditions, the phylum *Ascomycota* dominates among fungi, followed by the phylum *Basidiomycota*; *Aspergillus* is the predominant genus, consistent with oxygen-limited conditions. Under aerobic fermentation conditions, the dominant bacterial phylum is *Cyanobacteria*, with the genus *Cyanobacteria* being the most prevalent; under anaerobic fermentation conditions, the dominant bacterial phyla are *Firmicutes* and *Actinobacteria*, with the genera *Staphylococcus* and *Corynebacterium* being the most prevalent. Oxygen limitation and aerobic conditions significantly impact bacterial community composition but not fungal community composition.

Under aerobic conditions, the dominant bacterial genus is *Cyanobacteria*, with a relative abundance ranging from 79.09 to 38.75%; the genus *Bacillus* has a relative abundance ranging from 11.62 to 0.50%; the genus *Staphylococcus* has a relative abundance ranging from 11.62 to 0.50%. Under oxygen-limited conditions, the dominant bacterial genus is *Staphylococcus*, with a relative abundance ranging from 77.42 to 15.97%; the genus *Corynebacterium* has a relative abundance ranging from 44.55 to 2.94%. The dominant bacteria under aerobic fermentation conditions, such as the genera *Cyanobacteria* and *Bacillus*, are aerobic bacteria, while the dominant bacteria under anaerobic fermentation conditions, such as the genera *Staphylococcus* and *Corynebacterium*, are facultative anaerobes, and *Cyanobacteria* are absent (Table 2); this difference may be due to the lack of oxygen allowing *Staphylococcus* and *Corynebacterium* to replace *Cyanobacteria* and *Bacillus* as the dominant genera. Oxygen can influence bacterial community structure and succession to some extent. Anaerobic fermenters can limit oxygen concentration to lower levels, but in practice, vacuum pumps can be used to control oxygen concentration; experiments have shown that reducing oxygen content with vacuum pumps can cause significant differences in microbial community composition compared to aerobic conditions.

**Table 2:** Unique and shared bacteria under aerobic curing and oxygen-limited curing conditions.

Unique bacteria under aerobic curing conditions	Unique bacteria under oxygen-limited curing conditions	Shared bacteria
*Unclassified Cyanobacteria*	*Corynebacterium*	*Staphylococcus*
*Brevibacterium*	*Enteractinococcus*	*Pantoea*
*Unclassified Proteobacteria*	*Atopostipes*	*Aerococcus*
*Lactobacillus*	*Tetragenococcus*	*Pseudomonas*
*Unclassified Rhizobiaceae*		
*Bacillus*		

Under aerobic conditions, the dominant fungal genus is *Aspergillus* (Table 3), with a relative abundance ranging from 87.54 to 25.07%; under anaerobic conditions, the dominant fungal genus is *Aspergillus*, with a relative abundance ranging from 77.42 to 15.97%. *Aspergillus*, a saprophytic fungus, is one of the main factors causing mold during storage of cigar tobacco leaves. In the cigar industry production process, technicians often control the moisture content of cigar tobacco leaves and other processes to inhibit the reproduction of *Aspergillus* and reduce the risk of mold in cigar tobacco leaves. Therefore, the reduction in relative abundance of *Aspergillus* in this study is beneficial for controlling the rate of mold in cigars (Chattopadhyay et al., 2021; Ye et al., 2021).

**Table 3:** Unique and shared fungi under aerobic curing and oxygen-limited curing conditions.

Unique fungi under aerobic curing conditions	Unique fungi under oxygen-limited curing conditions	Shared fungi
*Fusarium*	*Xeromyces*	*Aspergillus*
*Vishniacozyma*	*Gibberella*	*Cladosporium*
*Mrakia*	*Colletotrichum*	*Alternaria*
	*Penicillium*	*Wallemia*
	*Golubevia*	*Sampaiozyma*
		*Stemphylium*

#### 3.1.3 Microbial community composition under three oxygen-limited conditions

**Figure 3:**
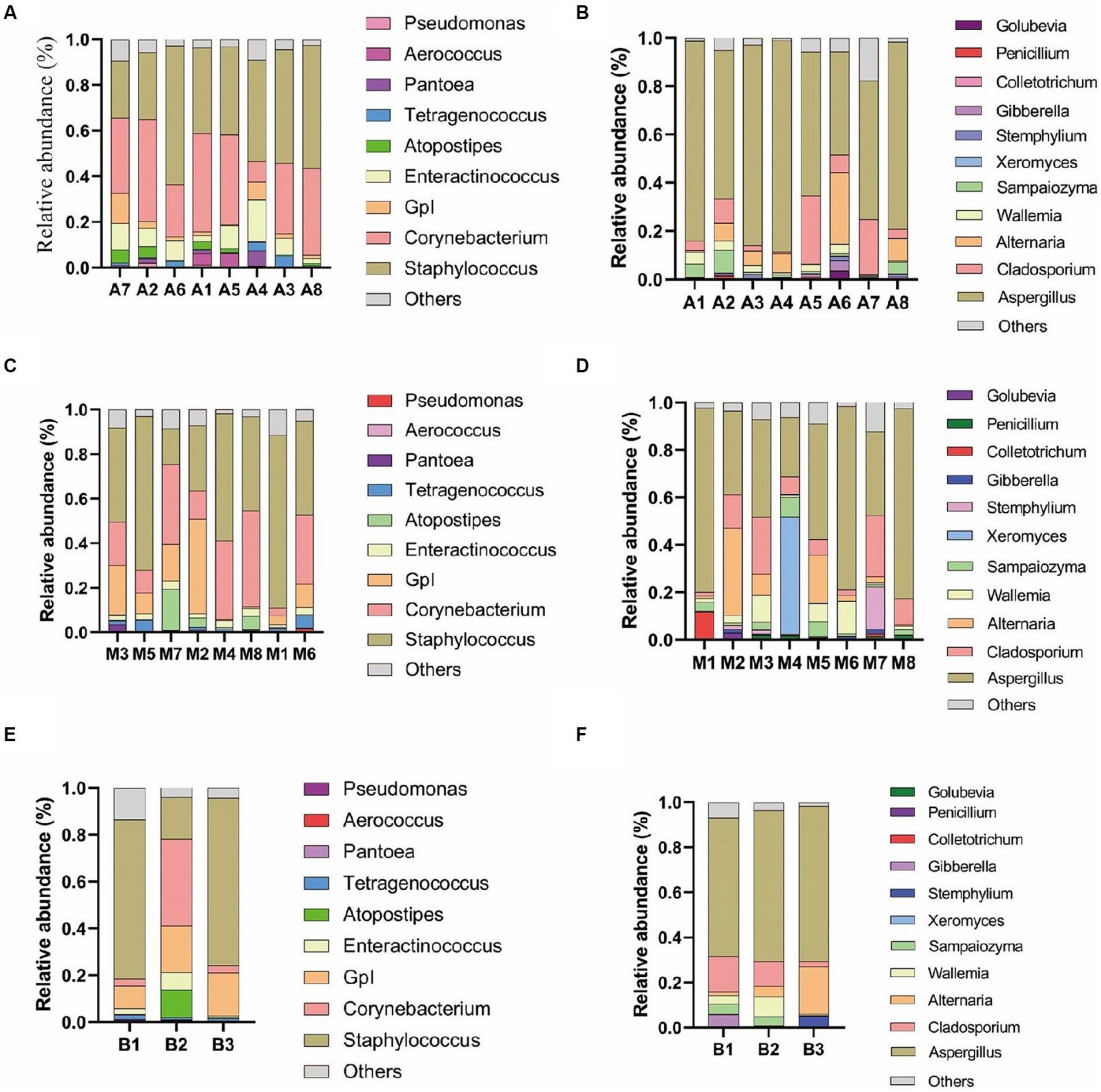
Shows the main bacterial genera **(A)** and fungal genera **(B)** in cigar samples under 0.1% oxygen concentration; the main bacterial genera **(C)** and fungal genera **(D)** under 6 to 12% oxygen concentration; and the main bacterial genera **(E)** and fungal genera **(F)** under 15% oxygen concentration.

As depicted in Figure 3, under 0.1% oxygen limitation conditions, the dominant bacterial genera were *Staphylococcus* and *Corynebacterium*, with *Staphylococcus* having a relative abundance ranging from 60.77 to 24.82%, and *Corynebacterium* ranging from 44.55 to 9.06%. The dominant fungal genus was *Aspergillus*, with a relative abundance ranging from 87.54 to 42.85%; *Enteractinococcus* ranged from 11.59 to 2.40%. Under 6 to 12% oxygen limitation conditions, the dominant bacterial genera remained *Staphylococcus* and *Corynebacterium*, with *Staphylococcus* ranging from 77.42 to 15.97%, and *Corynebacterium* from 42.94 to 3.53%. The dominant fungal genus was again *Aspergillus*, with a relative abundance ranging from 80.17 to 25.07%; *Enteractinococcus* ranged from 3.87 to 1.17%. Under 15% oxygen limitation conditions, the dominant bacterial genera were still *Staphylococcus* and *Corynebacterium*, with *Staphylococcus* ranging from 71.51 to 17.86%, and *Corynebacterium* from 36.99 to 2.94%. The dominant fungal genus remained *Aspergillus*, with a relative abundance ranging from 68.89 to 61.35%; *Enteractinococcus* ranged from 7.38 to 0.67%. The dominant microbial genera under the three oxygen limitation conditions were consistent and ubiquitous; however, the 0.1% oxygen limitation condition was more conducive to the enrichment of the genus *Enteractinococcus*.

### 3.2 Microbial community diversity under three oxygen limitation conditions

**Figure 4:**
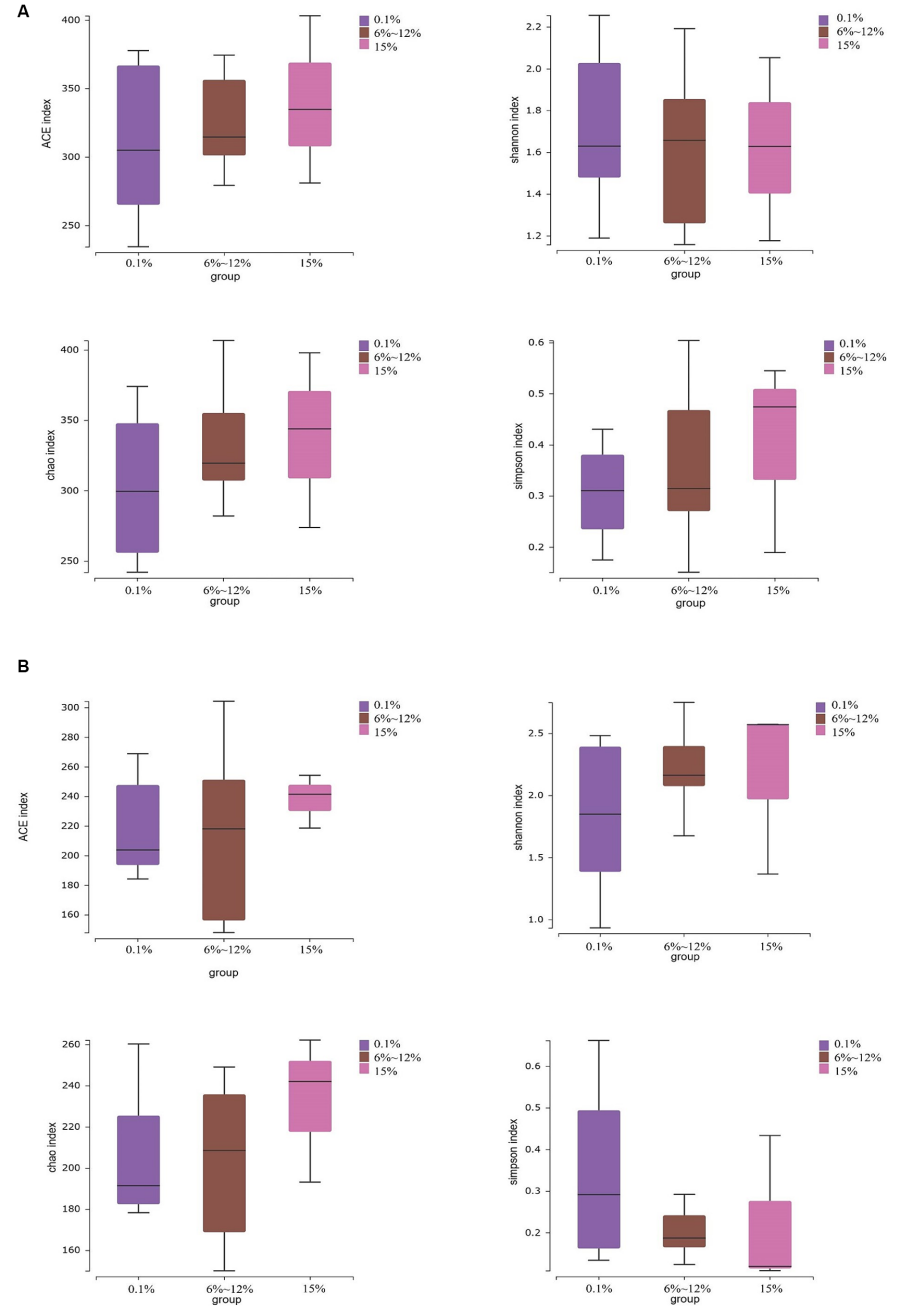
Alpha diversity of bacterial communities **(A)** and alpha diversity of fungal communities **(B)** in cigar tobacco leaf samples fermented at oxygen concentrations of 0.1, 6–12%, and 15%.

**Figure 5:**
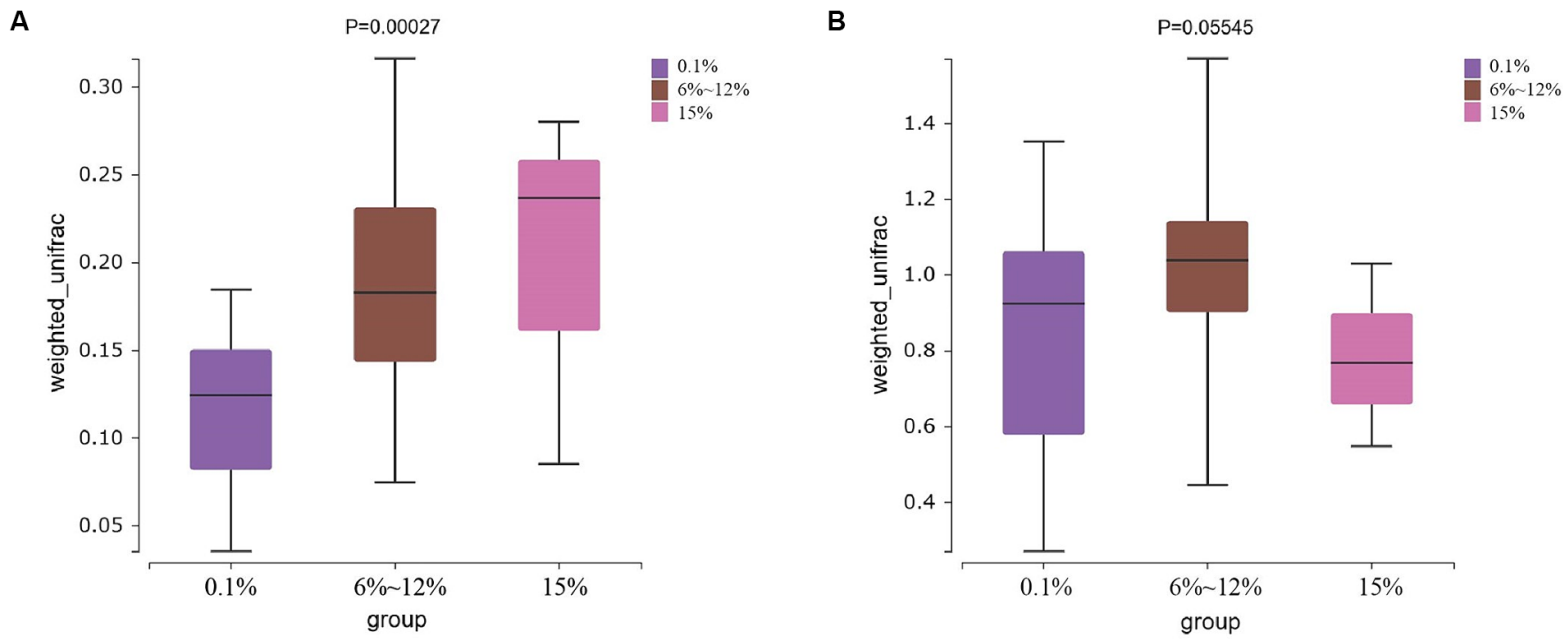
Beta diversity of bacterial communities **(A)** and beta diversity of fungal communities **(B)** in cigar samples fermented at oxygen concentrations of 0.1, 6–12%, and 15%.

**Figure 6:**
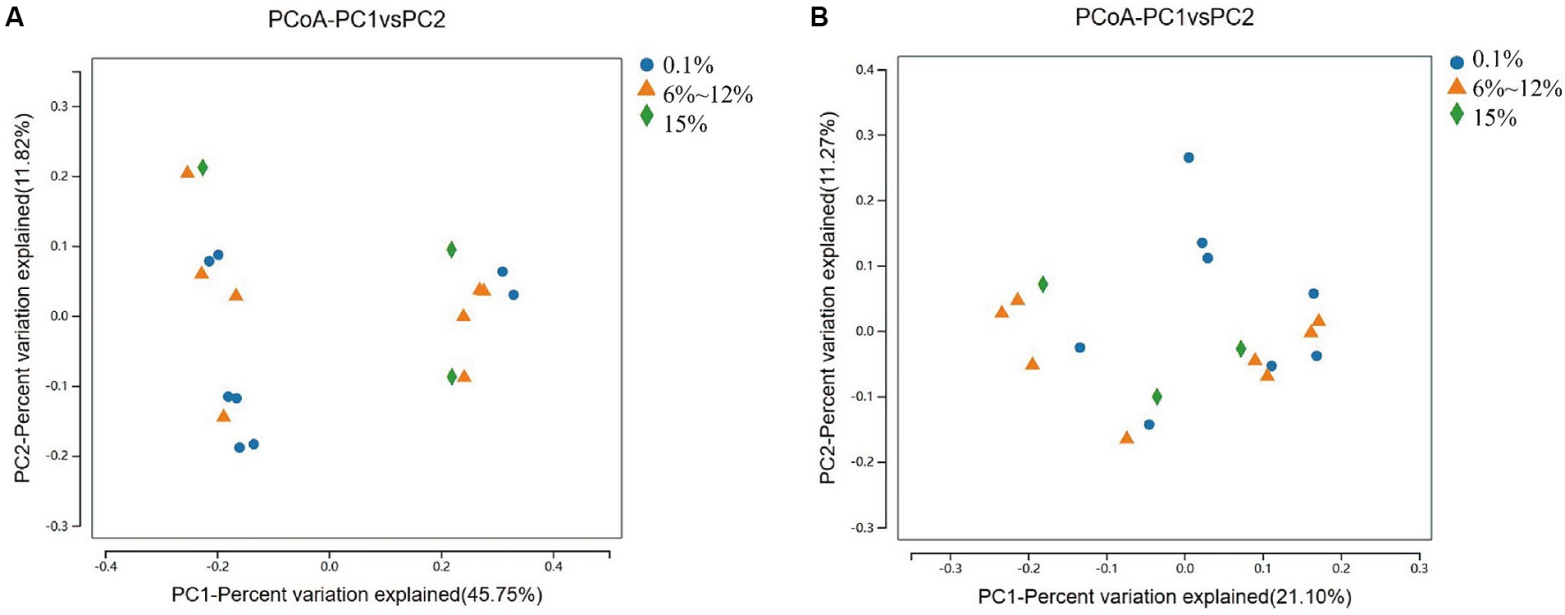
PCoA analysis of bacterial communities **(A)** and PCoA analysis of fungal communities **(B)** in cigar samples fermented at oxygen concentrations of 0.1, 6–12%, and 15%.

An analysis of the microbial diversity in cigar tobacco leaves was conducted to explore the differences in microbial communities under three fermentation methods with controlled oxygen concentrations of 0.1, 6–12%, and 15%. The richness of microbial communities in cigar tobacco leaf samples under different fermentation methods was measured using α-diversity indices based on Chao1, ACE, Simpson, and Shannon indices. Α-diversity indices reflect the richness and diversity of bacterial communities during the artificial fermentation process of cigar tobacco leaves. Beta-diversity based on the Bray-Curtis distance matrix was used to measure the differences in species composition of microbial communities in cigar tobacco leaf samples under different fermentation methods. As shown in Figure 4, the α-diversity from high to low under the three oxygen restriction conditions is in the order of 15% oxygen restriction, 6–12%, and 0.1%, indicating that higher oxygen concentrations lead to greater richness and diversity of bacterial communities. As shown in Figure 5, there are significant differences in β-diversity of bacterial communities under the three oxygen restriction conditions, while there are no significant differences in β-diversity of fungal communities, indicating that different oxygen restriction conditions have a greater impact on the species composition of bacterial communities than on fungal communities. Principal Coordinate Analysis (PCoA) is a multivariate data analysis method used to visualize similarities or differences between samples. In PCoA analysis, samples that cluster together indicate similar community compositions; samples with the same fermentation time generally cluster together, while there is a greater degree of dispersion among other samples, indicating that the microbial community in cigar tobacco leaves is constantly changing and varies greatly during fermentation. As shown in Figure 6, bacterial communities exhibit 45.75% variation along the horizontal axis and 11.82% variation along the vertical axis due to oxygen restriction conditions; fungal communities exhibit 21.10% variation along the horizontal axis and 11.27% variation along the vertical axis due to oxygen restriction conditions. The variation caused by fermentation methods is smaller for fungi than for bacteria.

**Figure 7:**
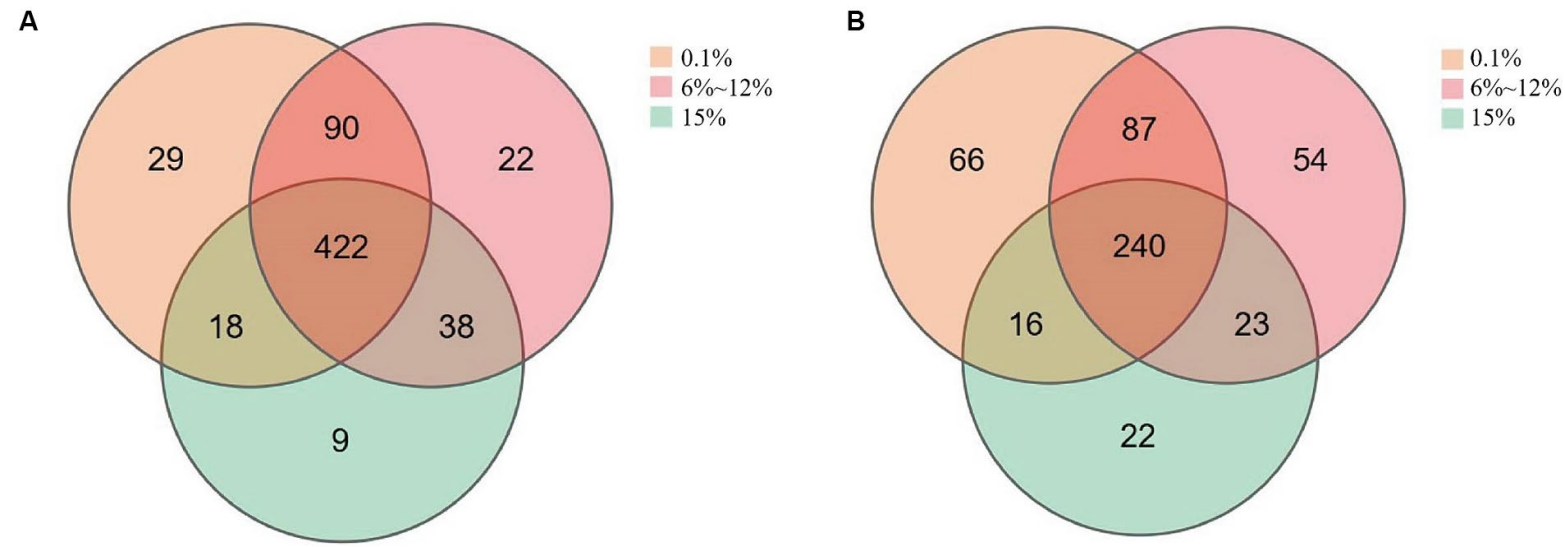
The number of unique and shared bacteria **(A)** and fungi **(B)** in cigar samples fermented at oxygen concentrations of 0.1, 6–12%, and 15%.

Venn diagrams were utilized to enumerate both the shared and unique microorganisms within the microbial communities of cigar tobacco leaves fermented under different oxygen concentrations, to better quantify the differences in microbial communities present in cigar tobacco leaves fermented at varying levels of oxygen. As shown in Figure 7, there are 422 bacterial species common to cigar tobacco leaves under all three oxygen-limiting conditions. Among these, the leaves fermented at oxygen concentrations of 0.1 and 6 to 12% share a greater number of similar bacterial species, while those fermented at an oxygen concentration of 15% have the fewest unique bacterial species. There are 240 fungal species common to cigar tobacco leaves under all three conditions. Similarly, leaves fermented at 0.1 and 6 to 12% oxygen concentrations share a greater number of similar fungal species, while those fermented at an oxygen concentration of 15% have the fewest unique fungal species. Different oxygen concentrations may promote the enrichment of specific microorganisms.

**Figure 8:**
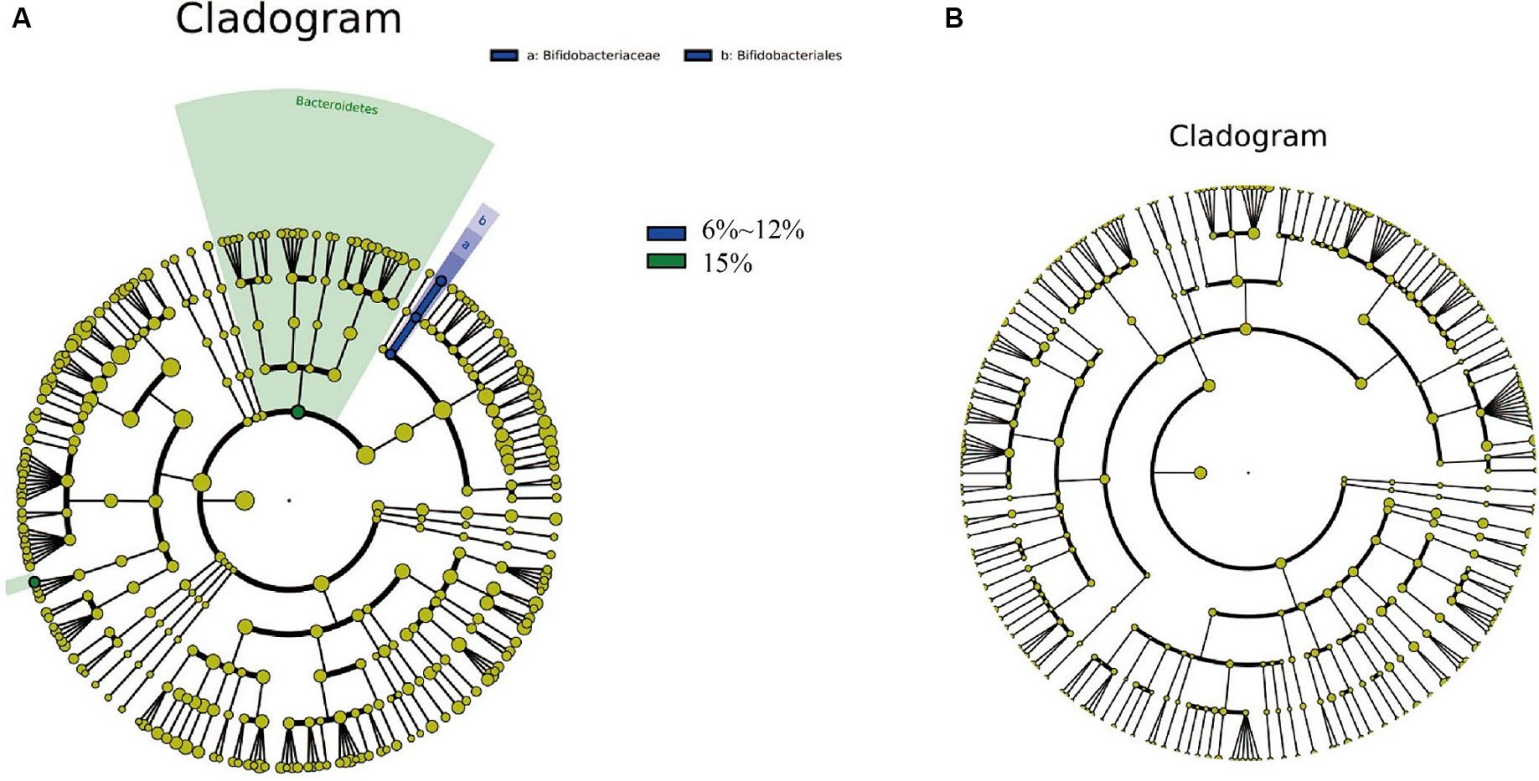
Unique and shared bacteria **(A)**, as well as unique and shared fungi **(B)** in cigar samples fermented at oxygen concentrations of 0.1, 6–12%, and 15%.

To explore the differences in cigar tobacco leaves fermented at oxygen concentrations of 0.1%, 6–12, and 15%, Linear discriminant analysis Effect Size (LEfSe) analysis was conducted, revealing significant differences below the phylum level (Figure 8). LEfSe can be compared between multiple groups using Python to identify biomarkers with statistically significant differences between different groups. The circles from inside to outside represent the classification of bacteria and fungi from phylum to genus, with each group’s corresponding color indicating significantly different bacterial taxonomic groups. The differential species in bacteria from cigar samples fermented at different oxygen concentrations include one phylum, one order, one family, and two genera (Figure 8A); there were no specific fungi in cigar samples fermented at different oxygen concentrations (Figure 8B).

**Figure 9:**
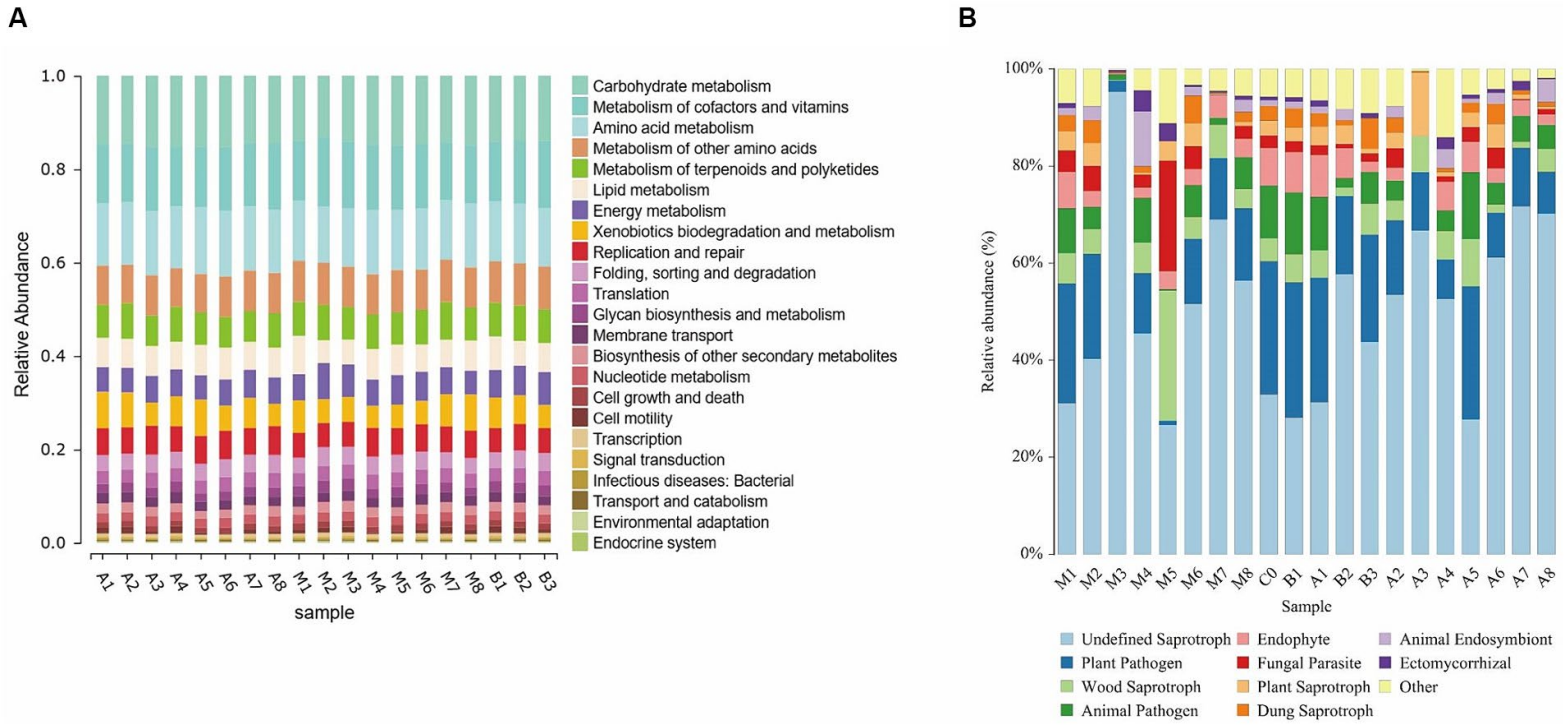
KEGG functional prediction **(A)** and fungal functional gene prediction **(B)**.

Using the PICRUSt2 analysis platform, functional predictions were made based on 16S rRNA amplicon sequencing data by matching the obtained OTU abundance table with the Kyoto Encyclopedia of Genes and Genomes (KEGG) database, linking species and their functions to obtain a rough distribution of the overall community function (Liu et al., 2021). As shown in Figure 9A, bacterial functional genes in cigar tobacco leaves are mainly enriched in the degradation of harmful compounds, biosynthesis of beneficial metabolites, photosynthesis, nitrogen metabolism, and energy metabolism. *Staphylococcus*, being a dominant group, reduces colony diversity and may inhibit the growth of bacteria that primarily express related genes, resulting in an overall lower abundance of these genes.

The FUNGuild database was used to classify identified fungal OTUs. The results indicate that as fermentation progresses, the functional diversity of the fungal community in tobacco leaves decreases (Figure 9), with fungi that have pathogenic and symbiotic nutritional modes gradually diminishing, including animal pathogens, plant pathogens, and endophytic fungi. However, the proportion of saprotrophic fungi gradually increases in cigar tobacco leaves, accounting for more than 50% of the total fungal population.

### 3.3 Microbial-chemical relationships

#### 3.3.1 Analysis of conventional chemical components in cigar tobacco leaves under oxygen-limiting conditions

The contents of six chemical components—starch, reducing sugars, total sugars, alkaloids, total nitrogen, chlorine, and potassium—were analyzed (Supplementary Table S1). Fermentation time significantly impacted the starch, total nitrogen, and total sugar content of the cigar tobacco leaves. As fermentation time increased, the content of starch and total nitrogen in the tobacco leaves showed a gradual decreasing trend, while the content of reducing sugars and total sugars initially increased and then decreased. The alkaloid content did not fluctuate significantly during fermentation.

Studies have indicated that during the fermentation process of cigar tobacco leaves, the contents of total sugars, reducing sugars, total nitrogen, and proteins decrease as fermentation time extends (Guo et al., 2022; Zhang et al., 2023). In this study, the contents of reducing sugars and total sugars first increased and then decreased during fermentation. The primary reason is that the oxygen-limiting conditions restricted bacterial growth and metabolism in the early stages of fermentation. Fungi decomposed starch into monosaccharides and polysaccharides, providing conditions for bacterial growth and metabolism, leading to an initial increase followed by a decrease in the content of reducing sugars and total sugars; it has been reported that *Aspergillus* and *Staphylococcus* produce enzymes necessary for starch degradation, namely debranching α-amylase, which leads to an increase in total sugar content and provides nutrients for subsequent microbial growth (De Mot and Verachtert, 1985; Lakshmi et al., 2013; Jia et al., 2020). The main volatile components during the fermentation process of cigar tobacco leaves are nicotine and solanesol. Previous research has shown that the nicotine content in artificially fermented Yunnan cigar tobacco leaves is about 4.4 to 6.6%, with a decreasing trend during fermentation and a relatively rapid rate of change (Wu et al., 2023). In this study, the nicotine content in the analyzed cigar tobacco leaf samples was approximately 3.4 to 3.9%, with little fluctuation during fermentation.

The composition and content of chemical components in tobacco leaves are closely related to the quality of the leaves. Previous research has found that nicotine significantly impacts strength and taste; soluble sugar content plays an obvious role in enhancing smoking quality, and monosaccharide content is a key factor affecting the Maillard reaction. An increase in monosaccharide content during fermentation can promote increased hygroscopicity and softer texture of tobacco leaves. Organic acids in tobacco leaves not only regulate the acid–base balance, enhance smoke richness, and improve smoking quality but also coordinate smoke concentration and enhance overall evaluation (Ma et al., 2022). Changes in the chemical composition of tobacco leaves, the composition and changes of surface microbial communities, as well as their interactions, will ultimately affect the quality of tobacco leaves.

**Figure 10:**
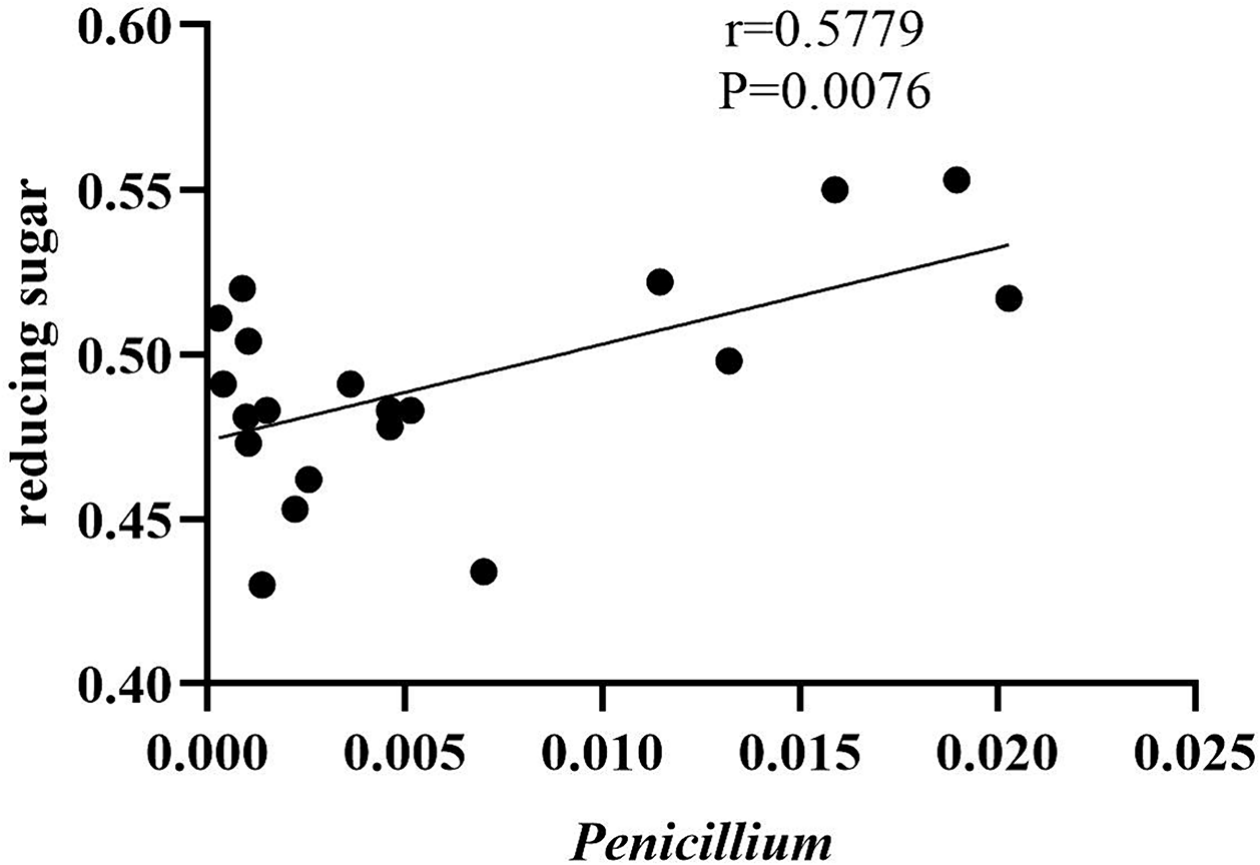
Correlation between reducing sugars and *Penicillium*.

As shown in Figure 10, the content of reducing sugars was positively correlated with the genus *Penicillium*, suggesting that fungi from this genus may have decomposed certain carbohydrates, thereby promoting the generation of reducing sugars. Literature indicates that *Penicillium* possesses a complex enzyme system, including amylase, cellulase, and protease, which can break down substances in tobacco leaves into sugars, amino acids, and other components (Li et al., 2019). There was no significant relationship between total alkaloids, total sugars, total nitrogen content, and the fungal or bacterial communities.

#### 3.3.2 Precursors of tobacco flavor components – amino acids

Microorganisms act on the internal components of tobacco leaves, as shown in Table 4. Their metabolism can produce a variety of active enzymes such as α-amylase, protease, pectinase, and cellulase, breaking down macromolecules in the leaves into small molecules that can generate aroma. These small molecules can also activate various enzyme systems, accelerating the transformation of aromatic substances (Li et al., 2003; Jianjun et al., 2006; Porter et al., 2016).

**Table 4:** Types and functions of related biological enzymes during tobacco leaf fermentation.

Types of biological enzymes	Decompose or transform substrates	Product
Protease	Protein	Amino acid
Amylase	Starch	Dextrin, maltose, glucose
Maltase	Maltose	Glucose
Invertase	Sucrose	Glucose, fructose
Cellulase	Cellulose	Glucose
Pectinase	Pectin	Soluble sugar
Lipase	Triacylglycerol	Monoacylglycerol, diacylglycerol, free fatty acids
Polyphenol oxidase	Phenolic substances	Quinone substances
Lipoxygenase	Carotenoids	Geraniol, violet ketone, purple yellow aldehyde

The proteins in tobacco leaves are continuously broken down by microbial action, producing short-chain fatty acids. The ketone compounds produced by fatty acids further interact and undergo the Maillard reaction. The Maillard reaction allows organic substances that are not primarily composed of sugars to develop a more diverse color and odor after maturation. This series of actions begins with the interaction between carbohydrate molecules and amino acids. They first form an unstable transitional structure, which then produces hundreds of different byproducts through further reactions, resulting in a brown appearance and rich, robust aroma. The color and odor changes in cigars are directly related to the Maillard reaction. The odors produced by the Maillard reaction are more complex and flavorful than those produced by caramelization. In this process, nitrogen and sulfur atoms from amino acids react with compounds containing carbon, hydrogen, and oxygen to create new types of molecules, adding layers to the aroma. The Maillard reaction also produces pyrazines associated with the aroma of popcorn (Long-jie et al., 2020).

**Figure 11:**
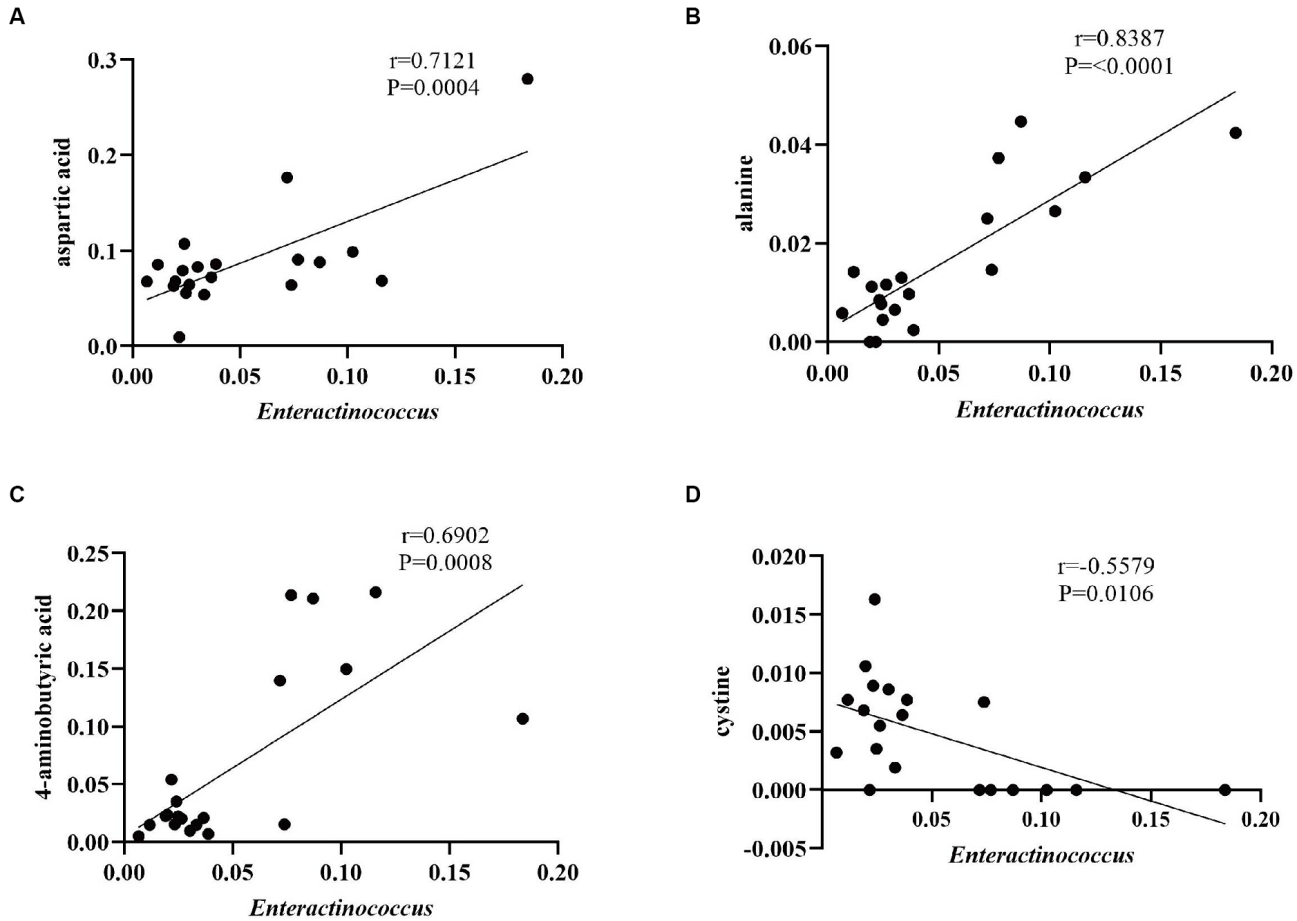
Correlation between aspartic acid and *Enteractinococcus* **(A)**; correlation between alanine and *Enteractinococcus* **(B)**; correlation between 4-aminobutyric acid and *Enteractinococcus* **(C)**; and correlation between cysteine and *Enteractinococcus* **(D)**.

As shown in Figure 11, the genus *Enterococcus* showed a positive correlation with aspartic acid, alanine, and 4-aminobutyric acid, and a negative correlation with cysteine. The genus *Enterococcus* can secrete lipases during fermentative metabolism to break down substances in tobacco leaves into organic acids, which are then transformed into amino acids under the action of enzymes (Chen et al., 2015). Amino acids are the raw materials for the synthesis of proteins, the degradation products of proteins, and the precursors for the synthesis of nicotine, polyphenols, and other substances, which contribute greatly to the carbon and nitrogen metabolism and tobacco leaf quality in tobacco plants (Supplementary Table S2). They undergo non-enzymatic browning reactions with carbohydrate compounds and are an important source of flavor substances in tobacco leaves (Li et al., 2018). The Maillard reaction of aspartic acid and alanine with glucose has a good flavor enhancement effect on tobacco (Sheng et al., 2011).

### 3.4 Changes in organoleptic quality under three oxygen restriction conditions

As shown in Supplementary Table S3, compared with the uncured tobacco leaves, the scores of various sensory evaluation indexes (mellowness, richness, miscellaneous gas, fullness, fluency, tuff, and balance) of cured tobacco were improved. The sensory scores of the three oxygen restriction conditions were 6–12%, 15, and 0.1% from high to low. The relative abundance of staphylococci in the three oxygen restriction conditions was 6–12%, 15, and 0.1% in descending order. The results indicated that a decrease in oxygen concentration led to a decrease in the relative abundance of *Staphylococcus*, ultimately leading to a decline in the sensory quality of cigar tobacco leaves. The greatest differences in sensation were richness and sweetness; under the condition of an oxygen concentration of 6–12%, the cigar leaf score was higher than that of the other two conditions. This may be due to the fact that the relative abundance of *Pseudomonas* was higher than that of the other two groups. *Pseudomonas* can degrade macromolecular organic compounds in tobacco leaves and reduce nicotine content (Vishwakarma and Verma, 2021).

## 4 Conclusion

This study examined the microbial community composition, diversity, and patterns of change during the artificial fermentation process of cigar tobacco leaves, as well as their association with changes in chemical components across four fermentation cycles and 20 samples. The main conclusions obtained are as follows:

1. Aerobic conditions and oxygen-limited conditions have a significant differential impact on bacterial community composition but not on fungal community composition; under aerobic conditions, the main bacteria are *Cyanobacteria* and Bacillus, while under oxygen-limited conditions, the main bacteria are *Staphylococcus* and rod-shaped Bacillus.
2. The three oxygen-limited conditions have a significant differential impact on bacterial community composition but not on fungal community composition; 0.1% oxygen limitation is more conducive to the enrichment of Enterococcus. Although the microbial communities of fermented cigar tobacco leaf samples differ under oxygen-limited conditions, the dominant genera are the same and universal, mainly including *Staphylococcus*, rod-shaped Bacillus, and *Aspergillus*.
3. Under oxygen-limited conditions, Enterococcus occupies a certain advantage and shows a positive correlation with aspartic acid, alanine, and 4-aminobutyric acid, and a negative correlation with cysteine.

Currently, there is a scarcity of research on the dynamic changes of microbes during the fermentation process of cigar tobacco leaves, and the dynamic changes in chemical components caused by microbes have not been widely studied. Therefore, further research can focus on the key microbes during the fermentation process of cigar tobacco leaves, their changes and functions, as well as the relationship between key microbes and chemical components. This will lay the foundation for elucidating the mechanism of action of microbes in tobacco leaf fermentation and promoting the industrialization of targeted microbial fermentation of cigar tobacco leaves.

## Data availability statement

The original sequence data reported in this article has been deposited in the genome sequence archives of the National Center for Biotechnology Information in the United States under accession numbers PRJNA1055691 and PRJNA1055872, and is publicly accessible at: https://www.ncbi.nlm.nih.gov.

## Author contributions

JY: Conceptualization, Methodology, Writing – original draft. FX: Writing – review & editing, Supervision. DL: Methodology, Writing – review & editing. JC: Investigation, Writing – review & editing. GSh: Validation, Writing – review & editing. GSo: Project administration, Writing – review & editing. YL: Project administration, Writing – review & editing.

## Footnotes

^1^ https://meta.bgi.com/
