# Supplementary material for: Oxygen regulation of microbial communities and chemical compounds in cigar tobacco curing
Source: Front Microbiol. 2024 Jul 23;15:1425553. doi: 10.3389/fmicb.2024.1425553 (PMC11300322; doi:10.3389/fmicb.2024.1425553)
Supplement: Supplementary file 1 [file Table_1.DOCX]

Supplementary Material

Oxygen Regulation of Microbial Communities and Chemical Compounds in Cigar Tobacco Curing

Juan Yang^134^, Fang Xue^2^, Dongliang Li^2^, Jiaowen Chen^2^, Guiyang Shi^134^, Guangfu Song^2*^, Youran Li^134*^,（Co-Corresponding author）

* Correspondence: Youran Li: [liyouran@jiangnan.edu.cn](mailto:liyouran@jiangnan.edu.cn); Guangfu Song: [songguangfu@126.com](mailto:songguangfu@126.com)

# Supplementary Data

The original sequence data reported in this article has been deposited in the genome sequence archives of the National Center for Biotechnology Information in the United States under accession numbers PRJNA1055691 and PRJNA1055872, and is publicly accessible at https://www.ncbi.nlm.nih.gov.

# Supplementary Tables

**Supplementary Table 1. Content of Conventional Chemical Components in Cigar Tobacco Leaves under Oxygen-Limiting Conditions**

| Serial number(Dry basis %) | Total plant alkaloids | Chlorine | Potassium | Total sugars | Reducing sugar | Total nitrogen | Starch |
| --- | --- | --- | --- | --- | --- | --- | --- |
| A1 | 3.516 | 1.857 | 4.085 | 0.923 | 0.473 | 8.909 | 12.760 |
| A2 | 3.528 | 1.724 | 3.996 | 1.084 | 0.498 | 9.039 | 13.071 |
| A3 | 3.555 | 1.878 | 3.929 | 1.085 | 0.520 | 9.256 | 11.583 |
| A4 | 3.591 | 1.723 | 3.910 | 1.000 | 0.504 | 8.872 | 11.919 |
| A5 | 3.681 | 1.767 | 4.365 | 0.876 | 0.434 | 8.726 | 12.524 |
| A6 | 3.572 | 1.787 | 3.999 | 1.023 | 0.483 | 8.353 | 12.975 |
| A7 | 3.621 | 1.824 | 3.859 | 0.962 | 0.478 | 9.023 | 12.024 |
| A8 | 3.663 | 1.930 | 3.977 | 0.971 | 0.483 | 8.396 | 12.056 |
| B1 | 3.697 | 2.177 | 3.437 | 1.373 | 0.483 | 8.076 | 14.013 |
| B2 | 3.649 | 1.716 | 3.918 | 0.975 | 0.491 | 8.175 | 12.206 |
| B3 | 3.397 | 2.011 | 4.363 | 0.948 | 0.491 | 8.297 | 9.464 |
| C0 | 3.653 | 1.861 | 4.147 | 0.928 | 0.453 | 9.287 | 12.691 |
| M1 | 3.727 | 1.928 | 4.166 | 0.920 | 0.430 | 8.497 | 10.406 |
| M2 | 3.822 | 1.891 | 4.008 | 0.997 | 0.481 | 8.781 | 10.213 |
| M3 | 3.578 | 1.820 | 4.054 | 0.970 | 0.553 | 8.517 | 9.357 |
| M4 | 3.547 | 1.718 | 3.864 | 1.071 | 0.550 | 8.617 | 9.873 |
| M5 | 3.779 | 1.738 | 4.180 | 0.912 | 0.462 | 8.979 | 11.110 |
| M6 | 3.859 | 1.876 | 3.938 | 1.062 | 0.511 | 8.549 | 11.204 |
| M7 | 3.654 | 2.083 | 3.963 | 1.070 | 0.522 | 8.019 | 10.338 |
| M8 | 3.558 | 1.848 | 3.997 | 0.899 | 0.517 | 9.095 | 10.685 |

**Supplementary Table 2. Amino Acid Content in Tobacco Leaves**

| serial number(mg/g) | aspartic acid | alanine | valine | isoleucine | leucine | phenylalanine |
| --- | --- | --- | --- | --- | --- | --- |
| A1 | 0.107056 | 0.007719 | 0 | 0.045259 | 0.062084 | 0.079339 |
| A2 | 0.090729 | 0.037269 | 0.014152 | 0.00747 | 0.025251 | 0.035241 |
| A3 | 0.176706 | 0.025019 | 0 | 0.011547 | 0.017731 | 0.01947 |
| A4 | 0.279835 | 0.042396 | 0 | 0.00862 | 0.032003 | 0.043944 |
| A5 | 0.09862 | 0.02652 | 0.021506 | 0 | 0 | 0 |
| A6 | 0.087856 | 0.044695 | 0.003965 | 0.012549 | 0.029595 | 0.027365 |
| A7 | 0.068447 | 0.033427 | 0 | 0.017286 | 0.026069 | 0.022134 |
| A8 | 0.009447 | 0 | 0 | 0 | 0.058218 | 0.074686 |
| B1 | 0.055701 | 0.004527 | 0.012474 | 0.005166 | 0.00871 | 0.064777 |
| B2 | 0.063874 | 0.014626 | 0.013188 | 0.007659 | 0.000186 | 0.044864 |
| B3 | 0.067573 | 0.005818 | 0.00852 | 0.088066 | 0 | 0.030955 |
| C0 | 0.063023 | 0 | 0.020386 | 0.005301 | 0.009812 | 0.037798 |
| M1 | 0.085446 | 0.014205 | 0.033254 | 0.012554 | 0.017031 | 0.04593 |
| M2 | 0.067901 | 0.011151 | 0.029603 | 0.009661 | 0.018716 | 0.085315 |
| M3 | 0.079225 | 0.008535 | 0.002265 | 0.008891 | 0.013928 | 0.067409 |
| M4 | 0.082975 | 0.006528 | 0.00273 | 0.014137 | 0.005949 | 0.130721 |
| M5 | 0.064408 | 0.011559 | 0.028158 | 0.007049 | 0.01147 | 0.03716 |
| M6 | 0.054014 | 0.013028 | 0.021758 | 0.007407 | 0.01385 | 0.055356 |
| M7 | 0.085837 | 0.002449 | 0.017508 | 0.003124 | 0.006106 | 0.007651 |
| M8 | 0.072052 | 0.009691 | 0.026451 | 0.006695 | 0.011306 | 0.027378 |

**Supplementary Table 3. Sensory Evaluation Form for Cigar Smoke Style Characteristics**

| serial number | mellowness | richness | miscellaneous gases | fullness | fluency | finesse | sweetness | irritation | sensation of recirculation | Combus-tibility | grey | tuff | balance | score |  |
| --- | --- | --- | --- | --- | --- | --- | --- | --- | --- | --- | --- | --- | --- | --- | --- |
| C0 | 6 | 6 | 6 | 6 | 6 | 6 | 6 | 6 | 6 | 7 | 7 | 7 | 6 | 81 |  |
| B1 | 6 | 7 | 6 | 6 | 6.5 | 6 | 6 | 6 | 6 | 6.5 | 6.5 | 7 | 6 | 82 |  |
| B2 | 7 | 7.5 | 7 | 7 | 7 | 7 | 7 | 7 | 7 | 7.5 | 7.5 | 7.5 | 7 | 93 |  |
| B3 | 6.5 | 7 | 6.5 | 7 | 7 | 6 | 6 | 6.5 | 6 | 7.5 | 7 | 7 | 6.5 | 87 |  |
| A1 | 6 | 6.5 | 6.5 | 6.5 | 6 | 6 | 6 | 5.5 | 6 | 7 | 7 | 7 | 6 | 82 |  |
| A2 | 6 | 6 | 6.5 | 6 | 6 | 6 | 6 | 6 | 6 | 7 | 7 | 7 | 6 | 82 |  |
| A3 | 6 | 6 | 6 | 6 | 6 | 6 | 6 | 6 | 5.5 | 7 | 7 | 7 | 6 | 81 |  |
| A4 | 6 | 5.5 | 6 | 6 | 6 | 6 | 6 | 6 | 5.5 | 7 | 7 | 7 | 6 | 80 |  |
| A5 | 6 | 6 | 6.5 | 6 | 6 | 6 | 6 | 6 | 6 | 7 | 7 | 7 | 6 | 82 |  |
| A6 | 6 | 6 | 6.5 | 6 | 6 | 6 | 6 | 6 | 6.5 | 7 | 7 | 7 | 6 | 82 |  |
| A7 | 6 | 6 | 6 | 6.5 | 6 | 6 | 6 | 5.5 | 6 | 7 | 7 | 7 | 6 | 81 |  |
| A8 | 6 | 6.5 | 6 | 6.5 | 6 | 6 | 5.5 | 5.5 | 6 | 7 | 7 | 7 | 6 | 81 |  |
| M1 | 6 | 7 | 7 | 7 | 7 | 7 | 7 | 7 | 6 | 7.5 | 7.5 | 7.5 | 7 | 91 |  |
| M2 | 7 | 7.5 | 7.5 | 7 | 7 | 7 | 7 | 7 | 7 | 7.5 | 7.5 | 7.5 | 7 | 94 |  |
| M3 | 7 | 7.5 | 7 | 7 | 7 | 7 | 7 | 7 | 6 | 7.5 | 7.5 | 7.5 | 7 | 92 |  |
| M4 | 7 | 7.5 | 7 | 7 | 7 | 7 | 7 | 7 | 6 | 7.5 | 7.5 | 7.5 | 7 | 92 |  |
| M5 | 6.5 | 7.5 | 6.5 | 7 | 7 | 7 | 7 | 7 | 6.5 | 6.5 | 7.5 | 7.5 | 6.5 | 90 |  |
| M6 | 6.5 | 7 | 6.5 | 7 | 7 | 7 | 7 | 7 | 6 | 6.5 | 7.5 | 7.5 | 6.5 | 89 |  |
| M7 | 7 | 7.5 | 7 | 7 | 7 | 7 | 7 | 7 | 7 | 7.5 | 7.5 | 7.5 | 7 | 93 |  |
| M8 | 7 | 7.5 | 7 | 7 | 7 | 7 | 7 | 7 | 7 | 7.5 | 7.5 | 7.5 | 7 | 93 |  |
